# Feasibility and efficacy of shared decision making for first-admission schizophrenia: a randomized clinical trial

**DOI:** 10.1186/s12888-017-1218-1

**Published:** 2017-02-06

**Authors:** Mio Ishii, Yasuyuki Okumura, Naoya Sugiyama, Hana Hasegawa, Toshie Noda, Yoshio Hirayasu, Hiroto Ito

**Affiliations:** 1 Department of Psychiatry, Yokohama City University School of Medicine, 3-9 Fukuura, Kanazawa-ku, Yokohama, 236-0004 Japan; 2 Research Department, Institute for Health Economics and Policy, Association for Health Economics Research and Social Insurance and Welfare, 1-5-11 Nishishimbashi, Minato-ku, Tokyo, 105-0003 Japan; 3 Numazu Chuo Hospital, 24-1 Nakase-cho, Numazu, Shizuoka 410-8575 Japan; 4 Department of Social Psychiatry, National Center of Neurology and Psychiatry, 4-1-1 Ogawa-Higashi, Kodaira, Tokyo, 187-8502 Japan

**Keywords:** Shared decision making, SDM, Schizophrenia, Randomized controlled trial, Pilot study, Inpatient treatment

## Abstract

**Background:**

The feasibility of shared decision making (SDM) for patients with schizophrenia remains controversial due to the assumed inability of patients to cooperate in treatment decision making. This study evaluated the feasibility and efficacy of SDM in patients upon first admission for schizophrenia.

**Methods:**

This was a randomized, parallel-group, two-arm, open-label, single-center study conducted in an acute psychiatric ward of Numazu Chuo Hospital, Japan. Patients with the diagnosis of schizophrenia upon their first admission were randomized into a SDM intervention group or a usual treatment group in a 1:1 ratio. The primary outcome was patient satisfaction at discharge. The secondary outcomes were attitudes toward medication at discharge and treatment continuation at 6 months after discharge.

**Results:**

Twenty-four patients were randomly assigned. The trial was prematurely terminated due to slow enrollment. At discharge, the mean score on satisfaction was 23.7 in the SDM group and 22.1 in the usual care group (unadjusted mean difference: 1.6; 95% CI: −5.2 to 2.0). Group differences were not observed in attitude toward medication and treatment continuation. There was no statistically significant difference between the groups for the mean Global Assessment of Functioning score at discharge or length of stay as safety endpoint.

**Conclusions:**

No statistical differences were found between the SDM group and usual care group in the efficacy outcomes and safety endpoints. Large trials are needed to confirm the efficacy of the SDM program upon first admission for schizophrenia.

**Trial registration:**

The study has been registered with ClinicalTrials.gov as NCT01869660 (registered 27 May, 2013).

## Background

Patient-centered care, where patients and health service providers establish partnerships to ensure every decision for patients’ needs and preferences, has been a core component of modern general medicine [1, 2]. Shared decision making (SDM) is a model in which patients and clinicians collaborate together throughout the entire decision-making process, with information shared and patients allowed to state their individual preferences [3]. SDM is expected to embody the idea of patient-centered care in clinical medicine. A meta-analysis found that, compared with usual care, SDM improves a patients’ knowledge acquisition, confidence, and active participation in treatment [4].

On the other hand, there has been some controversy among psychiatrists regarding the feasibility of SDM in regards to psychiatric treatment, particularly when it involves the treatment of severe mental illnesses such as schizophrenia, with respect to patient vulnerability to paternalism and coercive treatment due to patient symptoms such as disorganized thinking and excessive suspicion [5, 6]. Therefore, SDM had not targeted patients with severe mental illness such as schizophrenia because of presumed inability to cooperate in treatment decision making. Along with the recovery movement, mental healthcare providers are now encouraged to support every patient in pursuing their life goals [7]. In addition, clinical guidelines [8, 9] now advocate the use of SDM in psychiatry as a patient-centered care.

Nevertheless, there is a lack of evidence of SDM in patients with severe mental illness. To the best of our knowledge, only five randomized controlled trials have assessed the efficacy of SDM in patients with schizophrenia and related disorders [10–14]. Furthermore, there are serious limitations with the previous studies. First, only two trials [10, 11] focused on inpatient treatments in which coercive treatments are commonly administered. Second, all trials, with the exception of one that assessed long-term adherence and readmission rates [11], used only soft outcomes such as treatment satisfaction, perceived involvement in treatment decision making, and participants’ feelings of empowerment. Third, no study focused a homogenous sample of patients who do not have a history of psychiatric hospitalization. We believe it is important to focus on patients without prior experience of hospitalization, because better relationships between patients and medical providers in the early treatment phase for schizophrenia lead to better compliance [15].

Additional trials are needed to establish the feasibility and efficacy of SDM in patients with schizophrenia. We aimed to evaluate the feasibility and efficacy of SDM in patients upon first admission for schizophrenia.

## Methods

### Design overview

Details of the study protocol have been reported elsewhere [16]. The objective of this study was to evaluate the feasibility and efficacy of an SDM intervention compared to usual treatment on patient satisfaction at discharge and treatment continuation 6 months post-discharge for first-admission patients with schizophrenia. This was an investigator-initiated, single-center, randomized trial in which participants were randomly assigned either SDM plus usual care or usual care. Assessments were completed at admission, discharge, and 6 months after discharge. The trial was registered at ClinicalTrials.gov (NCT01869660) on May 27, 2013, and the first patient was recruited on June 4, 2013, and the last patient completed follow-up on September 29, 2015. The present study was originally planned to recruit 58 patients but was stopped prematurely due to slow patient enrollment. The institutional review board of the Yokohama City University, Japan, approved this study (No.: A130321008). All participants provided written informed consent or, if the participant was younger than 20 years of age, the legal guardian provided written informed consent.

### Setting and participants

Participants were consecutively recruited from an acute psychiatric ward of the Numazu Chuo Hospital, located in Numazu city, Shizuoka, Japan. Numazu Chuo Hospital is the only psychiatric hospital that receives emergency admissions for a population of 870,115 inhabitants. We assessed the following eligibility criteria within 3 days of admission: patients were aged 16–65 years; had no history of psychiatric admission (first admission); had a diagnosis of schizophrenia spectrum disorder (including schizophrenia, schizotypal, and delusional disorders defined according to the diagnosis codes F20–F29 in the International Statistical Classification of Diseases, 10th revision [ICD-10]). Exclusion criteria included moderate to profound mental retardation, organic mental disorders (ICD-10 codes: F00–F09), inability to converse in Japanese, and severe conceptual disorganization (a score of 5 or more on the Brief Psychiatric Rating Scale) [17, 18].

### Randomization

Randomization was conducted by the central web-based randomization system after obtaining written informed consent. Participants were randomly assigned in a 1:1 ratio using a computer-generated random number sequence. Assignment was balanced for the stratification factors of sex, age (younger or older than 20 years of age), and onset of illness (less or more than 1 year) by using a minimization method.

### Interventions

In the SDM group, participants received the SDM model program in addition to usual psychiatric inpatient care. The SDM model program is a 15–20-min weekly intervention during the acute psychiatric ward stay, and its development has been detailed previously [16]. The intervention consists of three sequential elements: assessing patient’s perceptions on their on-going treatments by a self-report questionnaire; sharing patients’ and medical staffs’ perceptions on the treatments in a 15–20-min meeting; and patients together with medical staff deciding on a care plan for the next week. As a medical team, a primary physician, a primary nurse, and others participated in the meetings. To improve adherence and quality of the intervention, independent supervisors managed intervention schedules, facilitated meetings, and educated medical staff.

In the usual care group, participants received usual psychiatric inpatient care, which is mainly pharmacological treatment. During the hospitalization, primary physicians examined their patients every day and nurses helped patients by focusing on self-care activities and the daily activity program. Primary physicians and nurses usually discussed the patient’s overall progress and plan for discharge. However, there was no fixed occasion for the patient and staff to share all the information. In addition, there was no certainty that patients would be introduced to the concept of SDM or that they would be empowered to actively participate in the treatment.

Patients in both SDM and usual care groups received treatments by the same medical staff in the ward—15 primary psychiatrists, nurses and three psychiatric social workers.

### Outcomes and follow-up

The primary outcome was patient satisfaction at discharge. Patient satisfaction was assessed using the Japanese version of the Client Satisfaction Questionnaire (CSQ-8 J), a 8-item self-rated measure [19, 20]. The CSQ-8 J assesses overall satisfaction with care received using a four-point Likert scale. Scores range from 8 to 32, with higher values indicating greater satisfaction. The CSQ-8 J has good internal consistency (Cronbach’s α = .83) and moderate convergent validity (*r* = .36–.49) with the client satisfaction inquiry [21].

Secondary outcomes included attitudes toward medication at discharge and treatment continuation 6 months after discharge. Attitudes were assessed using the Japanese version of the Drug Attitude Inventory-10 (DAI-10), a 10-item self-rated measure [22, 23]. The DAI-10 assess attitude toward medication using a true-false scale. Scores range from −10 to 10, with higher values indicating more positive attitudes. The DAI-10 has good internal consistency (Cronbach’s α = .97) and adequate test-retest reliability (*r* = .81) [23]. Treatment continuation was assessed by identifying whether a patient received outpatient psychiatric treatment within 30 days prior to the follow-up time (i.e., at 6 months after discharge). We identified information on treatment continuation from medical records if the patient continued treatment at the Numazu Chuo Hospital or if not, by asking patients with a telephone call. Symptom severity, as assessed by the Brief Psychiatric Rating Scales (BPRS) at discharge, was removed as a secondary outcome as this outcome was incorrectly specified in the original study protocol (14). We also amended the protocol to include the Global Assessment of Functioning at discharge [24] and length of stay as safety endpoints.

### Statistical analysis

We estimated unadjusted mean difference (MD) and risk difference (RD) with their 95% confidence interval (95% CI) between the groups (SDM vs. usual care) using Student’s *t* test for continuous variables (i.e., patient satisfaction and attitude toward medication) and chi-squared test for a categorical variable (i.e., treatment continuation). We also calculated standardized MD and risk ratio with their 95% confidence interval between the groups. To control characteristics such as sex, age, duration of illness, and symptom severity assessed by the BPRS at admission, we estimated adjusted MD and RD using a marginal structural binomial model [25]. All data were analyzed by R version 3.2.0.

## Results

### Baseline characteristics

**Fig. 1:**
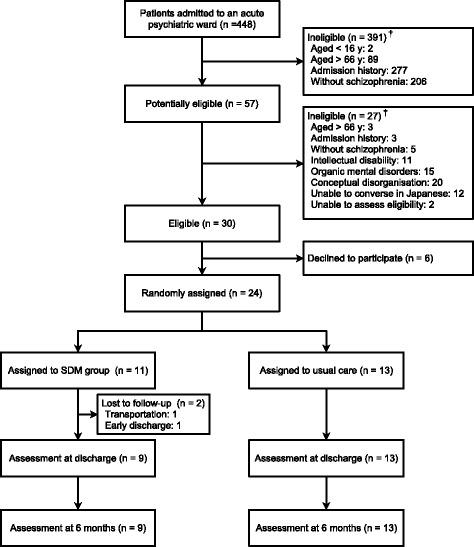
Flow diagram. SDM = shared decision making.†Some patients met 1 > criterion

A total of 448 patients were screened, of whom 24 were randomly assigned (Fig. 1). The most common reason for ineligibility was a prior history of psychiatric admission (*n* = 280). After randomization, two patients were lost before outcome assessments due to hospital transportation and early discharge without doctors’ permission. Table 1 shows baseline characteristics in the groups. In general (87% of total of 30 sessions), a primary doctor, a ward nurse, and a facilitator participated in the SDM meeting. The median number of sessions received was 3 (range: 2–5).

**Table 1:** Baseline characteristics

Characteristic	Total (*n* = 22)	SDM (*n* = 9)	Usual care (*n* = 13)
Women, *n* (%)	7 (31.8)	3 (33.3)	4 (30.8)
Mean age at admission (SD), y	39.1 (11.7)	41.6 (13.6)	37.4 (9.8)
At least 1-year after the onset of illness, *n* (%)	14 (63.6)	7 (77.8)	7 (53.8)
Suicidal ideation, *n* (%)			
Absent	8 (36.4)	3 (33.3)	5 (38.5)
Present	9 (40.9)	5 (55.6)	4 (30.8)
Unknown	5 (22.7)	1 (11.1)	4 (30.8)
Physical comorbidity, *n* (%)	6 (27.3)	4 (44.4)	2 (15.4)
Mean BPRS score at admission (SD)	57.0 (14.8)	49.6 (14.2)	62.2 (12.9)
Mean GAF score at admission (SD)	17.5 (12.5)	20.9 (17.1)	14.2 (6.4)
Average length of stay (SD), day	66.7 (40.4)	66.5 (17.4)	65.2 (50.5)

*BPRS* the Brief Psychiatric Rating Scale, *GAF* the Global Assessment of Functioning scale, *SDM* shared decision making

### Outcomes

For the primary and secondary outcomes, group differences were not observed in both unadjusted and adjusted analyses (Table 2). At discharge, the mean score on satisfaction was 23.7 in the SDM and 22.1 in usual care group (unadjusted MD: 1.6; 95% CI: −5.2 to 2.0). The mean score of attitudes toward medication was 3.8 in the SDM and 2.3 in usual care group (unadjusted MD: 1.5; 95% CI: −5.6 to 2.7). At 6 months after discharge, continuation rate was 89% in the SDM group and 69% in usual care group (unadjusted RD: 19.7; 95% CI: −12.8 to 52.1). There were no statistically significant differences between the groups for the mean Global Assessment of Functioning score at discharge or length of stay (Table 2).

**Table 2:** Primary and secondary outcomes

Outcome	SDM	Usual care	Crude treatment effect (95% CI)	Adjusted treatment effect (95% CI)^c^	Standardized mean difference/risk ratio (95% CI)
Primary outcome					
Mean satisfaction at discharge (SD)	23.7 (3.9)	22.1 (3.7)	1.6 (−5.2 to 2.0)^a^	−0.8 (−4.2 to 2.6)^a^	0.39 (−0.47 to 1.24)^d^
Secondary outcomes					
Mean attitude toward medication at discharge (SD)	3.8 (3.7)	2.3 (4.8)	1.5 (−5.6 to 2.7) ^a^	−1.1 (−4.4 to 2.3)^a^	0.31 (−0.55, 1.16)^d^
Treatment continuation at 6 months after discharge, n (%)	8 (88.9)	9 (69.2)	19.7 (−12.8 to 52.1)^b^	22.1 (−24.9 to 70.1)^b^	0.36 (0.05, 2.72)^e^
Harm					
Mean GAF score at discharge (SD)	55.6 (11.2)	47.8 (18.9)	7.7 (−23.1 to 7.7)	4.0 (−13.0 to 20.0)^a^	0.44 (−0.42, 1.30)^d^
Average length of stay (SD), day	66.7 (40.4)	66.5 (17.4)	1.3 (−39.6 to 37.0)	−1.7 (−43.6 to 40.9)^a^	0.03 (−0.82, 0.88)^d^

*BPRS* the Brief Psychiatric Rating Scale, *CI* confidence interval, *GAF* the Global Assessment of Functioning, *SDM* shared decision making

^a^Mean difference (95% CI) between the SDM and usual care groups

^b^Risk difference (95% CI) between the SDM and usual care groups

^c^Adjusted for sex, age, illness duration, and symptom severity assessed by the BPRS at admission

^d^Standardized mean difference (95% CI) between the SDM and usual care groups

^e^Risk ratio (95% CI) between the SDM and usual care groups

## Discussion

We examined whether our SDM intervention—a complex intervention including assessment of patient’s perception, regular meetings, and shared care planning—improved clinical outcomes for patients with schizophrenia in the early treatment stage. This pilot study is the first randomized controlled trial to evaluate efficacy of SDM targeting patients with first-admitted schizophrenia. We found no statistical differences between the SDM group and usual care group in the primary and secondary outcomes.

Until now, SDM for patients with acute psychosis was deemed difficult because of the patient’s decisional capacity, time constraints, and coercive atmosphere [26]. Consequently, medical providers have been hesitant about patient’s active involvement in decision making. However, in our study, treatment adherence was maintained throughout the trial. All cases in the intervention group could receive interventional SDM sessions, for 20 min per week, for multiple times in spite of acute symptoms and busyness at the emergency ward. There were no statistically significant differences between groups in safety endpoints. None of the participants dropped out from the SDM intervention, with the exception of hospital transportation and early discharge without a doctor’s permission. These results suggest the feasibility of SDM in patients with first-admission schizophrenia.

The strengths of our trial include a high rate of informed consent acquisition, assessment of treatment continuation after discharge as a hard endpoint, narrow eligibility criteria of first-admission schizophrenia, and homogeneous quality of interventions supported by the supervision team. However, this study had several limitations. First, the sample size was too small, as we had stopped the investigation early due to slow enrollment. As a result, our trial did not have adequate statistical power to detect a difference in primary and secondary outcomes. The primary reason for the slow enrollment was the narrow eligibility criteria focusing on only those whom it was their first admission for schizophrenia. Future studies should include a longer recruitment period, conduct a multi-center trial, or use wider eligibility criteria such as including patients with history of psychiatric hospitalization. Second, duration of follow-up might be too short. A cohort study of discharged patients with schizophrenia showed that the average time to discontinuation of antipsychotics after discharge was 289 days for olanzapine and 254 days for risperidone [27]. We might need to set the duration of follow-up to longer than 6 months to maximize statistical power. Third, the treatment intensity of SDM was limited due to not including peers and family in the intervention. Jönsson et al. [28] considered personal supporters to be essential members of SDM in the treatment of severe mental illness; furthermore, there have been some attempts in palliative care [29, 30]. Not providing a post-discharge program is another concern for the intensity. Fourth, there was a contamination risk because we conducted the trial in a ward where the same medical staff could be involved in both SDM and usual care groups. Thus, patients in usual care were treated by physicians who received education on the concept of SDM, although these patients never had an opportunity to receive the standardized SDM intervention. Lastly, we did not include any measure of SDM as outcome and any assessment of fidelity of the intervention; however, this limitation might not induce bias, because independent supervisors participated in regular meetings.

## Conclusions

This randomized controlled trial suggests that SDM intervention is feasible during acute inpatient treatment for schizophrenia. Large trials are needed to confirm the efficacy of the SDM in patients with schizophrenia.

## Abbreviations

BPRS: Brief psychiatric rating scale
CSQ-8 J: Client satisfaction questionnaire Japanese version
DAI-10: Drug attitude inventory
ICD-10: The international statistical classification of diseases, 10th revision
SDM: Shared decision making
